# Characterization and Genomic Analysis of a New Bacteriophage *Klebsiella pneumoniae* CTF-1 from Turkey

**DOI:** 10.3390/antibiotics14111153

**Published:** 2025-11-14

**Authors:** Kübra Can Kurt, Edip Tokuç, Halil Kurt, Duygu Nur Akın, Ahmet Sait, Sevcan Aydın, Mikael Skurnik, Hrisi Bahar Tokman

**Affiliations:** 1 Department of Basic Medical Sciences, Hamidiye Faculty of Dentistry, University of Health Sciences, Istanbul 34668, Turkey; 2 Medical Microbiology Department, Cerrahpasa Medical Faculty, Istanbul University-Cerrahpasa, Istanbul 34098, Turkey; edip.tokuc@ogr.iuc.edu.tr (E.T.); hrisib@iuc.edu.tr (H.B.T.); 3 Medical Biology Department, Hamidiye International School of Medicine, University of Health Sciences, Istanbul 34668, Turkey; halil.kurt@sbu.edu.tr; 4 Department of Genetics and Bioengineering, Nişantaşı University, Istanbul 34398, Turkey; darabaci@gmail.com; 5 Virology Laboratory, Pendik Veterinary Control Institute, Istanbul 34890, Turkey; ahmet.sait@tarimorman.gov.tr; 6 Biotechnology Section, Department of Biology, Faculty of Science, Istanbul University, Istanbul 34134, Turkey; sevcan.aydin@istanbul.edu.tr; 7 Human Microbiome Research Program, Department of Bacteriology and Immunology, Faculty of Medicine, University of Helsinki, 00290 Helsinki, Finland; mikael.skurnik@helsinki.fi

**Keywords:** *Klebsiella pneumoniae*, wound infection, phage therapy, antimicrobial resistance, whole-genome sequencing

## Abstract

**Background/Objectives:** *Klebsiella pneumoniae* is a clinically important pathogen that causes respiratory tract infections, pneumonia, wound infections, urinary tract infections, and sepsis. It is on the World Health Organization (WHO) priority pathogen list as it causes antimicrobial-resistant infections. The aim of this study was to isolate bacteriophages against pan-resistant *K. pneumoniae* isolated from clinical wound infections. **Results:** One of the isolated phages, CTF-1, possesses a linear double-stranded DNA genome that is 40,841 base pairs (bp) long and contains 44 predicted genes. Functional assignments were made for 31 of the predicted gene products, which are associated with genome replication, phage packaging, structural proteins, and host lysis, leaving 13 annotated as hypothetical proteins. Based on sequencing analysis, phage CTF-1 is a new member of the genus *Przondovirus* within the order *Autographivirales*. Phage CTF-1 was effective against 22 of 25 (88%) pan-resistant *K. pneumoniae* isolates. The latent period and lytic cycle of the phage were approximately 40 min, with a burst size of about 92 PFU/mL. **Conclusions:** Our findings suggest that *Klebsiella* phage CTF-1 is an excellent candidate for phage therapy due to its high lytic activity against pan-resistant *K. pneumoniae* strains and lack of genes encoding antibiotic resistance, toxins, virulence factors, or integrases.

## 1. Introduction

*K. pneumoniae* is a Gram-negative opportunistic pathogen belonging to the *Enterobacteriaceae* family. *K. pneumoniae* is ubiquitous in the environment and is regularly found in water, soil, animals, and plants. It is a causative agent of many infections, such as pneumonia, sepsis, wound infections, bacteremia, liver abscesses, and urinary tract infections [1,2,3,4]. *K. pneumoniae* may also cause pneumonia and sepsis, especially in immunosuppressed patients. Newborns, immunocompromised individuals, and elderly people are at the greatest risk of *K. pneumoniae* infections [5,6].

Wound infections cause many deaths worldwide every year. *K. pneumoniae*, *Staphylococcus aureus*, and *Pseudomonas aeruginosa* are the most prevalent causative agents of wound infections [7,8,9]. The emergence and alarmingly rapid global spread of multidrug-resistant (MDR) and pan-resistant *K. pneumoniae* strains are causing increased mortality. The WHO recognizes extended-spectrum β-lactamases (ESBLs) and carbapenem-resistant *K. pneumoniae* as global health threats, as these bacteria have become resistant to nearly all available antibiotics. *K. pneumoniae* is classified within the ESKAPEE (*Enterococcus faecium*, *Staphylococcus aureus*, *K. pneumoniae*, *Acinetobacter baumannii*, *Pseudomonas aeruginosa*, *Enterobacter species,* and *Escherichia coli*) group of pathogens due to its ability to ‘escape’ treatment [10,11,12,13,14]. The antibiotic colistin is used as a last resort in the treatment of multidrug-resistant *K. pneumoniae* [15,16]; however, it is proving less effective as the number of colistin-resistant *K. pneumoniae* strains increases [17]. Colistin-resistant *K. pneumoniae* strains are responsible for more than 90,000 infections and more than 7000 deaths per year in Europe alone [18].

The spread of MDR pathogens is indeed a major global problem that needs to be addressed. Bacteriophages (phages) are natural entities that infect and kill bacteria by lysis after proliferation. Phages, either alone or in combination with antibiotics, can be used to complement or replace antibiotics in various infections, a practice that is becoming increasingly widespread [19,20]. Phage therapy is promising for the treatment of multidrug-resistant Gram-negative bacilli. Phages are useful in therapy as they cause almost no side effects and can destroy biofilms [21,22,23]. Because of their high specificity against bacteria, phages can kill target bacterial strains without harming human cells or other beneficial bacteria [24,25,26]. Furthermore, phage therapy is effective against both antibiotic-resistant and antibiotic-sensitive pathogens. Phage therapy has also been shown to restore sensitivity to various antibiotics by disrupting antibiotic resistance mechanisms [27,28,29,30]. Thus, phages could be a potential clinical treatment, especially against antibiotic-resistant bacterial pathogens.

The aim of this study was to isolate and characterize a novel lytic bacteriophage against *K. pneumoniae* and investigate the effectiveness of phage CTF-1 on pan-resistant *K. pneumoniae* isolates.

## 2. Results

The *Klebsiella* phage CTF-1 was isolated from a wastewater sample and produced clear plaques on a lawn of *K. pneumoniae*. Based on genome analysis, CTF-1 can be classified within the genus *Przondovirus* in the subfamily *Studiervirinae* of the family *Autographivirales*, according to the latest criteria established by the International Committee on Taxonomy of Viruses [31,32].

### 2.1. Physiological Characterization of the Klebsiella Phage CTF-1

**Figure 1:**
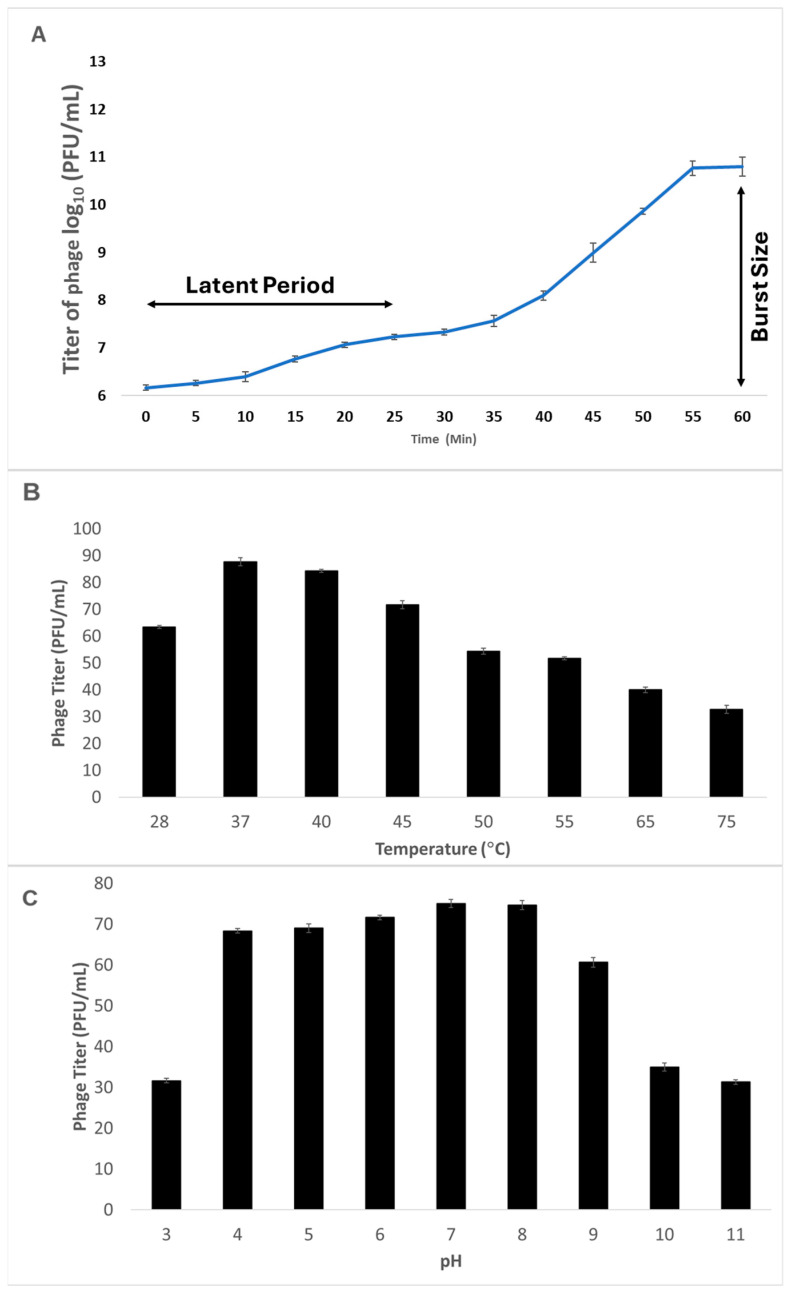
Biological characterization of the *Klebsiella* phage CTF1. (**A**) One-step growth curve analysis of the *Klebsiella* phage CTF1. (**B**) Thermal stability of the *Klebsiella* phage CTF1. (**C**) pH stability of the *Klebsiella* phage CTF1.

The latent period and lytic cycle of the phage were approximately 40 min, with a burst size of about 92 PFU per milliliter (Figure 1A). The optimum temperature for phage CTF-1 propagation was 37 °C (Figure 1B), and the optimal pH was between 7 and 8 (Figure 1C). Of the 25 *K. pneumoniae* strains isolated from wound samples, 22 (88%) were sensitive to CTF-1 and 3 (12%) were resistant.

The effectiveness of the *Klebsiella* phage CTF-1 phage has been tested against other *E. coli*, *Pseudomonas aeruginosa,* and *Staphylococcus aureus* strains; however, the phage has not demonstrated any effectiveness against these bacteria. In this study, we observed that *Klebsiella* phage CTF1 displayed a restricted host range, exhibiting lytic activity solely against *K. pneumoniae*.

### 2.2. Annotations and Characteristics of the Complete Genome of Klebsiella Phage CTF1

**Figure 2:**
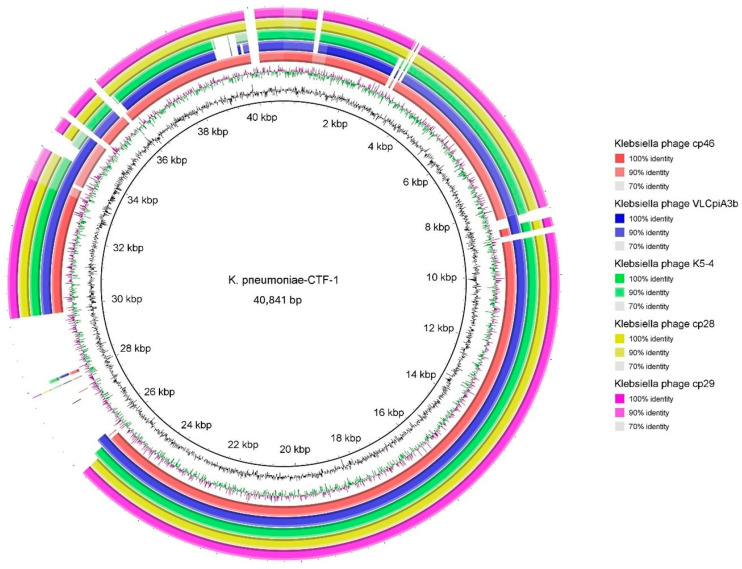
Circular map of the whole genome of *K. pneumoniae* CTF-1 and comparative genome maps of genome sequences of closely related bacteriophages. Gaps in the circles represent regions of low or no similarity.

**Figure 3:**
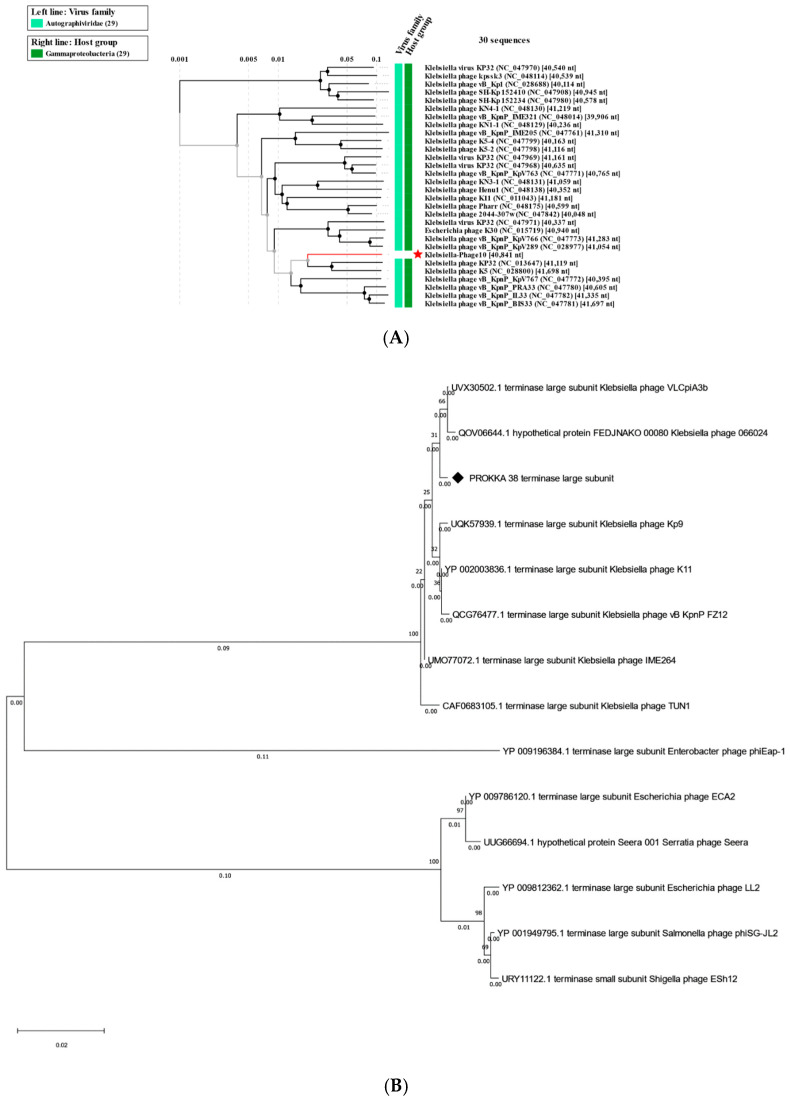
The evolutionary association of phage with closely related bacteriophages. (**A**) Phylogenetic trees of *K. pneumoniae* CTF-1 constructed from the whole-genome alignment generated by VipTree. The red asterix indicates the location of CFT-1. (**B**) Phylogenetic trees of the terminase large subunit from *K. pneumoniae* CTF-1 and closely related phages. The rhombus indicates the location of CTF-1 large terminase.

Third-generation long-read sequencing of the CTF-1 genome was performed using the Oxford Nanopore MinION. The phage has a linear double-stranded DNA genome of 40,841 bp in length with a 53.1% GC content. A circular map of the CTF-1 genome and genome maps of closely related bacteriophages are shown in Figure 2. Phage CTF-1 is most closely related (82.19% overall identity) to *Klebsiella* phage cp46 (OX335440.1) (Table 1). Therefore, *K. pneumoniae* CTF-1 is considered as a novel bacteriophage. The phylogenetic trees for phage CTF-1 based on both the terminase large subunit and whole-genome sequences are given in Figure 3.

**Figure 4:**
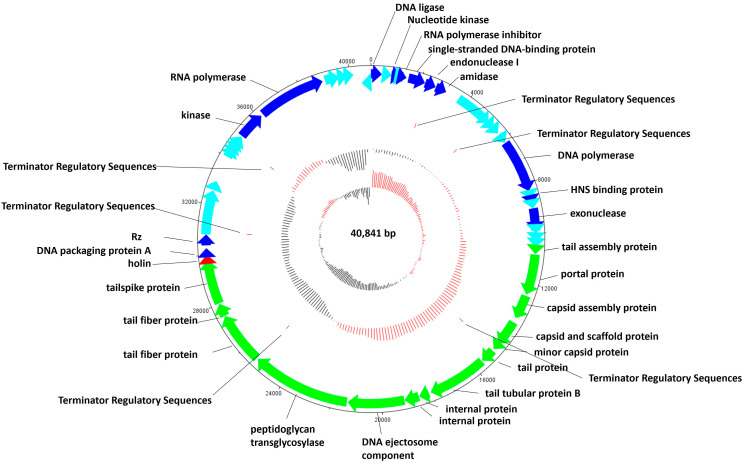
Genomic map of *K. pneumoniae* CTF-1 and its genetic characteristics. Structural proteins are represented in green, while genes associated with transcription and translation are in blue. Holins are shown in red, and hypothetical proteins in turquoise.

The genomic structure of phage CTF-1 is shown in Figure 4, showing a total of 49 predicted protein-coding open reading frames (ORFs). Based on BLASTp searches, functions were predicted for 44 predicted gene products as structural proteins or proteins with functions in replication or host lysis (Figure 4, Table S1). The remaining 13 ORFs were annotated as hypothetical proteins.

The Gp13 gene functions as a DNA polymerase and Gp45 as a DNA-dependent RNA polymerase. Gp6 functions as a single-stranded DNA-binding protein, while Gp07 and Gp17 were identified as an endonuclease and exonuclease, respectively (Table S1). Gp1, Gp3, Gp5, and Gp7 serve as other transcription- and replication-related proteins.

Lytic bacteriophages require enzymes such as endolysins, holins, and spanins for cell lysis. Among these, holins and spanins are responsible for cell lysis at the end of the phage growth cycle and disrupt the structure of the cell membrane via a transmembrane domain [33]. Gp35 is annotated as a holin, while Gp37 is identified as Rz, a spanin protein, both of which are involved in bacterial lysis (Figure 4). Gp35 and Gp37 share 100% and 98% identity, respectively. Gp35 carries a transmembrane domain between the amino acid residues 37 and 55 and can be classified as a Class II holin. The spanin-like protein G37 carries a transmembrane domain between 7 and 25.

The gene products Gp21–Gp34 are structural proteins involved in head and tail morphogenesis. Gp36 was annotated as the small terminase subunit and Gp38 as the large terminase subunit. The large terminase subunit is a conserved protein and therefore often used in the construction of phylogenetic trees (Figure 3B). Six rho-independent terminators were found in the *K. pneumoniae* CTF-1 genome (Table S2).

As no antibiotic resistance genes were identified in the phage CTF-1 genome, the phage can be considered as a safe therapeutic phage.

## 3. Discussion

The ever-increasing number of carbapenem-resistant and colistin-resistant *K. pneumoniae* strains poses a major global problem. For this reason, research and development of new phage-based therapies may help in the treatment of infections caused by resistant bacteria. In this study, a novel *Klebsiella* phage CTF-1 was isolated against *K. pneumoniae* strains obtained from clinical wound samples. The genome of CTF-1 was characterized and evaluated for its potential to be used as a therapeutic agent. The findings of the study provide a good basis for the development of phage-based therapies against *K. pneumoniae* strains isolated from clinical wound samples. According to the phage genome analysis, phage CTF-1 is classified as a new species in genus *Przondovirus* that belongs to the subfamily *Studiervirinae* in *Autographivirales*. This study showed that phage CTF-1 was able to infect 88% of the 25 clinical *K. pneumoniae* isolates. Since CTF-1 does not contain antibiotic resistance and toxin genes, it can be safely used in treatment. Forty-four gene products with functions as structural, genome replication or host lysis proteins were identified from the phage genome.

The presence of genes necessary for lysogenicity, such as integrase and excisionase enzymes, in a bacteriophage genome is the most important indicator of lysogenic characteristics [34]. Additionally, cloudy plaques and bacterial lawns are phenotypic indications of lysogenic character [35]. Our study concluded that the phage lacked lysogenic ability due to the absence of integrase and exonuclease enzymes in the genome, as well as the formation of clear plaques.

Wound infections are responsible for one-third of nosocomial infections and 70–80% of deaths from hospital-acquired infections. They are major agents in the development of mortality and morbidity in patients, particularly in developing countries [36,37]. Wound infections are difficult to diagnose and treat, are particularly common in hospitalized patients, and are likely to be infected with multiple pathogenic bacteria. Control of wound infections has become increasingly difficult due to the rising prevalence of infections caused by polymicrobial flora and widespread bacterial resistance to antibiotics. *E. coli*, *Klebsiella* spp., *S. aureus*, *P. aeruginosa*, and *Acinetobacter* spp. are the most common bacterial pathogens causing wound infections [38].

Bacteriophages are used in some countries to treat various bacterial infections, but standardized medical procedures have not yet been established. Due to lack of awareness among medical staff and the public, phage therapy has not yet become a generally accepted means of treatment. In order to utilize the enormous potential of phage therapy, it is necessary to choose the most effective bacteriophage [39,40]. The success of phage therapy relies on determining strategies to treat infections and decrease the outbreak of phage-resistant bacteria [41]. Today, phages have promising clinical applications in curing infections caused by antibiotic-resistant bacteria; however, phages still have restrictions in clinical application. As phage therapy research develops further over time, clinical applications of phages have a bright future.

The in vivo pharmacodynamics and pharmacokinetics of various phage products differ from those of antibiotics. Notably, purification and manufacturing of new phages are less costly than antibiotics. There are many differences in the clinical application of phages, because preparations that include various phages have a different biological profile.

Phage therapy has been applied against *K. pneumoniae* strains isolated from wound infections [42,43] and sepsis [44]. The KpJH46Φ2 phage was successfully used in combination with antibiotics in a patient with prosthetic joint infection caused by *K. pneumoniae* [42]. Yang, Wang, Zeng, Song, Zhang, Wei, Li, and Feng [43] isolated the *K. pneumoniae* RCIP0100 phage and demonstrated broad lytic activity against 15 of 27 MDR-KP strains. Similar to phage CTF-1, phage RCIP0100 was a promising candidate for phage therapy. The in vivo efficacy of *Klebisella* phages ɸKpBHU7, ɸKpBHU4, and ɸKpBHU14 was successfully tested in a mouse model of septicemia [44]. They efficiently lysed 71.42%, 77.14%, and 71.14% of clinical *K. pneumoniae* isolates, respectively. These results are comparable to the host range of phage CTF1.

Its in vitro activity against *K. pneumoniae* strains is critical for developing potential phage-based therapeutic agents. However, conducting in vitro animal studies and determining the pharmacokinetic and pharmacodynamic properties of the isolated phage are essential for the clinical application of phage therapy. The absence of in vivo studies in our work is a limitation of this study. Nevertheless, discovering new lytic phages against *K. pneumoniae* strains and molecularly characterizing these phages reveal candidates that could be used for phage therapy. For this reason, we plan to determine the in vivo therapeutic efficacy of the *K. pneumoniae* CTF-1 phage in future studies. We will evaluate the therapeutic efficacy of our phage in living organisms by inducing sepsis or pneumonia in a mouse model. In conclusion, phage CTF-1 can be considered as a safe and effective bacteriophage in the treatment of wound infections caused by multidrug-resistant *K. pneumoniae* strains.

## 4. Materials and Methods

### 4.1. Bacterial Strains and Culture Conditions

The 25 clinical *K. pneumoniae* strains used in the study were resistant to all antimicrobials, including colistin (pan-resistant), and were isolated from clinical wound specimens sent to the Cerrahpasa Medical Faculty, Medical Microbiology Laboratory from November 2019 to November 2020. The study was approved by the Clinical Research Ethics Committee of Istanbul University (83045809-604.01.02), and all the methods used met the guidelines and regulations. Bacteria culturing was performed in Tryptic Soy Broth (TSB) (Merck, Darmstadt, Germany) supplemented with 5 mM CaCl_2_ (Merck, Darmstadt, Germany) and MgCl_2_ (Merck, Darmstadt, Germany). Samples were inoculated on blood agar, chocolate agar, and MacConkey agar. Bacterial colonies were characterized using matrix-assisted laser desorption/ionization time-of-flight (MALDI-TOF) mass spectrometer (MS, Bruker, Rheinstetten, Germany). The disk diffusion method was performed in accordance with the guidelines of the European Committee on Antimicrobial Susceptibility Testing (EUCAST, Växjö, Sweden), and the results were evaluated according to EUCAST criteria. The disk diffusion method was used to determine the resistance profiles of *K. pneumonia* strains, a total of thirteen antibiotics were tested, including four cephalosporins, four penicillin derivatives, two aminoglycosides, and one each from the sulfonamide, fluoroquinolone, and polymyxin group antibiotics. Median zone diameters obtained for 13 antimicrobial agents were evaluated according to EUCAST guidelines and are presented in Table S3.

### 4.2. Isolation and Purification of Phage CTF-1

The isolation and purification of bacteriophages were carried out using the double-layer plate method. Activated sludge was collected for phage isolation from East Wastewater Treatment Plant (Bursa, Turkey) in November 2020. Wastewater samples were filtered through 0.2 μm syringe filters (Isolab, Istanbul, Turkey) to exclude bacteria. An amount of 50 μL *K. pneumoniae* was seeded in 2.5 mL of TSB medium supplemented with 5 mM MgCl_2_ and CaCl_2_ and incubated overnight at 37 °C. The cultures were centrifuged, and the supernatant was filtered again with a 0.22 μm filter to exclude bacterial cells. The filtrate was serially diluted with TSB from 10^−1^ to 10^−12^ and utilized in overlay agar plaque assays [19].

### 4.3. One-Step Growth Curve Analysis

A mixture of 0.1 mL phage with 9.9 mL of *K. pneumoniae* in TSB was incubated for 5 min at room temperature. The mixture was centrifuged for 5 min to remove free phages, and the bacterial pellet was resuspended in 10 mL TSB. Aliquots of 100 μL were withdrawn every 5 min for 60 min to determine phage titers. The number of phage progeny/number of latent infected cells determined the burst size [45]. Biological triplicate was applied to minimize variability.

### 4.4. Host Range Testing

The host range of the phage was determined using the spot test method. A total of 5 μL dilutions of phage were pipetted onto bacterial plates in soft agar. Petri dishes were left to dry for 1 h, then incubated upside down at 37 °C overnight. Plaque formation was investigated to record the ability of the phage to infect the host strain in question. The *K. pneumoniae* CTF-1 phage was also tested against *Escherichia coli*, *Pseudomonas aeruginosa*, and *Staphylococcus aureus* strains. During infectivity and host range assays, a test without phage was performed as a negative control.

### 4.5. Physical Stability of the Phage

The temperature stability of phage CTF-1 was determined by incubating the phage in TSB for 1 h at 4, 28, 37, 40, 45, 50, 55, 60, and 65 °C. The pH tolerance of the phage was tested by incubating the phage for 1 h in TSB adjusted to different pH values (2.0, 3.0, 4.0, 5.0, 6.0, 7.0, 8.0, 9.0, 10.0, 11.0, and 12.0). Phage titers were determined using the double-overlay assay. Each assay was carried out in biological triplicate, results were expressed as mean and standard deviation.

### 4.6. Isolation of Phage Genomic DNA

Phage particles were concentrated using PEG 6000 method. PEG 6000 (Merck, Darmstadt, Germany) was mixed into the phage solution to a final concentration of 10% (*w*/*v*), stirred overnight at +4 °C with gentle agitation (100 rpm), and then centrifuged at 12,000× *g* for 4 h. The supernatant was discarded, and the obtained pellet was dispersed into 200 μL of DNase- and RNase-free water. Phage genomic DNA was isolated using the Roche MagNA pure LC total nucleic acid isolation kit (Penzberg, Germany) in accordance with the manufacturer’s protocol. The quantity and quality of the phage DNA was measured with Thermo NanoDrop 2000c (Penzberg, Germany).

### 4.7. Phage Genome Sequencing with MinION™ and Bioinformatic Analysis

Genome sequencing was performed using the ONT ligation sequencing kit (SQK-LSK109, Oxford, UK) and the ONT native barcode kit (EXP-NBD104-114). The prepared libraries were loaded into an ONT MinION Flowcell v. 9.4.1 (FLO-MIN106D). Raw reads in Fast4 format were obtained using the MinKNOW program (v. 22.03.5). High-quality reads were obtained after barcodes and adapters were removed using the ONT-guppy (v. 6.0.6). The quality of raw fastq reads was controlled by FastQC v0.11.9 (https://www.bioinformatics.babraham.ac.uk/projects/fastqc/ (accessed on 1 June 2023)). Contigs were obtained using the flye de novo assembler (v. 2.8) [46]. BLAST v2.12.0 searches were performed against the NCBI nt reference database for taxonomic analysis of the contigs. Overall identity% of bacteriophage was calculated using the following formula: overall identity% = identity% × coverage [26]. The annotation of protein-coding genes was conducted by BLASTp against the NCBI nr database with an e value of 0.00001 and identity of 80% parameter after identifying the protein-coding genes using the prokka program v1.13 [47]. The existence of antibiotic resistance genes was checked using the deepARG program v1.0.2 [48], and rho-independent terminators were estimated using ARNold v1.0 [49].

## 5. Conclusions

The *K. pneumoniae* CTF-1 phage can be considered as a promising bacteriophage therapeutic candidate for wound infections caused by multidrug-resistant *K. pneumoniae* strains, because it does not contain toxins, integrase genes, or virulence factors.

## Figures and Tables

**Table 1:** The whole-genome sequence alignment of *K. pneumoniae*-CTF-1 against NCBI refseq database.

Scientific Name	Overall Identity%	Max Score	Total Score	Query Cover	E Value	Per. Ident	Acc. Len	Accession
Klebsiella phage cp46	82.20%	26,294	54,083	87%	0	94.48%	39,694	OX335440.1
Klebsiella phage VLCpiA3b	79.74%	25,398	50,989	86%	0	92.72%	40,231	ON602742.1
Klebsiella phage K5-4	78.20%	25,357	50,533	84%	0	93.10%	40,163	NC_047799.1
Klebsiella phage cp28	79.33%	25,236	51,261	85%	0	93.33%	39,661	OX335408.1
Klebsiella phage cp29	79.33%	25,236	51,261	85%	0	93.33%	39,662	OX335401.1

## Data Availability

The data underlying this article are available in GenBank accession number of PV550976.

## Supplementary Materials

The following supporting information can be downloaded at: https://www.mdpi.com/article/10.3390/antibiotics14111153/s1, Table S1. Annotation of the *K. pneumoniae* CTF-1 phage genome. Table S2. rho-independent terminator locations. Table S3. Tested antibiotics and mean zone diameters obtained for 25 *K. pneumoniae* strains.

## Abbreviations

The following abbreviations are used in this manuscript:

WHO	World Health Organization
MDR	Multidrug-resistant
ESBL	Extended-spectrum β-lactamases
ESKAPEE	*Enterococcus faecium*, *Staphylococcus aureus*, *Klebsiella pneumoniae*, *Acinetobacter baumannii*, *Pseudomonas aeruginosa*, *Enterobacter species*, and *Escherichia coli*
TSB	Tryptic soy broth
MALDI-TOF	Matrix-assisted laser desorption/ionization time-of-flight mass spectrometer
EUCAST	European Committee on Antimicrobial Susceptibility Testing
BLAST	Basic local alignment search tool
NCBI	National Center for Biotechnology Information
PEG	Polyethylene glycol
ORFs	Open reading frames

## References

[1] Effah C.Y., Sun T., Liu S., Wu Y. (2020). Klebsiella pneumoniae: An increasing threat to public health. Ann. Clin. Microbiol. Antimicrob..

[2] Lee C.-R., Lee J.H., Park K.S., Jeon J.H., Kim Y.B., Cha C.-J., Jeong B.C., Lee S.H. (2017). Antimicrobial resistance of hypervirulent Klebsiella pneumoniae: Epidemiology, hypervirulence-associated determinants, and resistance mechanisms. Front. Cell. Infect. Microbiol..

[3] Dai P., Hu D. (2022). The making of hypervirulent Klebsiella pneumoniae. J. Clin. Lab. Anal..

[4] Singh A.N., Singh A., Singh S.K., Nath G. (2024). Klebsiella pneumoniae infections and phage therapy. Indian J. Med. Microbiol..

[5] Guerra M.E.S., Destro G., Vieira B., Lima A.S., Ferraz L.F.C., Hakansson A.P., Darrieux M., Converso T.R. (2022). Klebsiella pneumoniae biofilms and their role in disease pathogenesis. Front. Cell. Infect. Microbiol..

[6] Xu L., Sun X., Ma X. (2017). Systematic review and meta-analysis of mortality of patients infected with carbapenem-resistant Klebsiella pneumoniae. Ann. Clin. Microbiol. Antimicrob..

[7] Mende K., Akers K.S., Tyner S.D., Bennett J.W., Simons M.P., Blyth D.M., Li P., Stewart L., Tribble D.R. (2022). Multidrug-resistant and virulent organisms trauma infections: Trauma infectious disease outcomes study initiative. Mil. Med..

[8] Yang S., Hu L., Zhao Y., Meng G., Xu S., Han R. (2024). Prevalence of multidrug-resistant bacterial infections in diabetic foot ulcers: A meta-analysis. Int. Wound J..

[9] Garry B., Samdavid Thanapaul R.J., Werner L.M., Pavlovic R., Rios K.E., Antonic V., Bobrov A.G. (2024). Antibacterial Activity of Ag^+^ on ESKAPEE Pathogens In Vitro and in Blood. Mil. Med..

[10] Holt K.E., Wertheim H., Zadoks R.N., Baker S., Whitehouse C.A., Dance D., Jenney A., Connor T.R., Hsu L.Y., Severin J. (2015). Genomic analysis of diversity, population structure, virulence, and antimicrobial resistance in Klebsiella pneumoniae, an urgent threat to public health. Proc. Natl. Acad. Sci. USA.

[11] Hung C.-H., Kuo C.-F., Wang C.-H., Wu C.-M., Tsao N. (2011). Experimental phage therapy in treating Klebsiella pneumoniae-mediated liver abscesses and bacteremia in mice. Antimicrob. Agents Chemother..

[12] Russo T.A., Marr C.M. (2019). Hypervirulent klebsiella pneumoniae. Clin. Microbiol. Rev..

[13] Bassetti M., Righi E., Carnelutti A., Graziano E., Russo A. (2018). Multidrug-resistant Klebsiella pneumoniae: Challenges for treatment, prevention and infection control. Expert Rev. Anti-Infect. Ther..

[14] Wyres K.L., Holt K.E. (2018). Klebsiella pneumoniae as a key trafficker of drug resistance genes from environmental to clinically important bacteria. Curr. Opin. Microbiol..

[15] Peng Q., Fang M., Liu X., Zhang C., Liu Y., Yuan Y. (2020). Isolation and characterization of a novel phage for controlling multidrug-resistant Klebsiella pneumoniae. Microorganisms.

[16] Pitout J.D., Laupland K.B. (2008). Extended-spectrum β-lactamase-producing Enterobacteriaceae: An emerging public-health concern. Lancet Infect. Dis..

[17] Aris P., Robatjazi S., Nikkhahi F., Marashi S.M.A. (2020). Molecular mechanisms and prevalence of colistin resistance of Klebsiella pneumoniae in the Middle East region: A review over the last 5 years. J. Glob. Antimicrob. Resist..

[18] Wyres K.L., Lam M.M., Holt K.E. (2020). Population genomics of Klebsiella pneumoniae. Nat. Rev. Microbiol..

[19] Townsend E.M., Kelly L., Gannon L., Muscatt G., Dunstan R., Michniewski S., Sapkota H., Kiljunen S.J., Kolsi A., Skurnik M. (2021). Isolation and characterization of Klebsiella phages for phage therapy. Ther. Appl. Res..

[20] Akturk E., Oliveira H., Santos S.B., Costa S., Kuyumcu S., Melo L.D., Azeredo J. (2019). Synergistic action of phage and antibiotics: Parameters to enhance the killing efficacy against mono and dual-species biofilms. Antibiotics.

[21] Sulakvelidze A., Alavidze Z., Morris J.G. (2001). Bacteriophage therapy. Antimicrob. Agents Chemother..

[22] Eskenazi A., Lood C., Wubbolts J., Hites M., Balarjishvili N., Leshkasheli L., Askilashvili L., Kvachadze L., van Noort V., Wagemans J. (2022). Combination of pre-adapted bacteriophage therapy and antibiotics for treatment of fracture-related infection due to pandrug-resistant Klebsiella pneumoniae. Nat. Commun..

[23] Karaiskos I., Galani I., Papoutsaki V., Galani L., Giamarellou H. (2022). Carbapenemase producing Klebsiella pneumoniae: Implication on future therapeutic strategies. Expert Rev. Anti-Infect. Ther..

[24] Nikolich M.P., Filippov A.A. (2020). Bacteriophage therapy: Developments and directions. Antibiotics.

[25] Pirnay J.-P., Djebara S., Steurs G., Griselain J., Cochez C., De Soir S., Glonti T., Spiessens A., Vanden Berghe E., Green S. (2024). Personalized bacteriophage therapy outcomes for 100 consecutive cases: A multicentre, multinational, retrospective observational study. Nat. Microbiol..

[26] Kurt K.C., Kurt H., Tokuç E., Özbey D., Arabacı D.N., Aydın S., Gönüllü N., Skurnik M., Tokman H.B. (2025). Isolation and characterization of new lytic bacteriophage PSA-KC1 against Pseudomonas aeruginosa isolates from cystic fibrosis patients. Sci. Rep..

[27] Al-Ishaq R.K., Skariah S., Büsselberg D. (2020). Bacteriophage treatment: Critical evaluation of its application on World Health Organization priority pathogens. Viruses.

[28] Gholizadeh O., Ghaleh H.E.G., Tat M., Ranjbar R., Dorostkar R. (2024). The potential use of bacteriophages as antibacterial agents against Klebsiella pneumoniae. Virol. J..

[29] Bozidis P., Markou E., Gouni A., Gartzonika K. (2024). Does phage therapy need a pan-phage?. Pathogens.

[30] Kapoor A., Mudaliar S.B., Bhat V.G., Chakraborty I., Prasad A.S.B., Mazumder N. (2024). Phage therapy: A novel approach against multidrug-resistant pathogens. 3 Biotech.

[31] Lefkowitz E.J., Dempsey D.M., Hendrickson R.C., Orton R.J., Siddell S.G., Smith D.B. (2018). Virus taxonomy: The database of the International Committee on Taxonomy of Viruses (ICTV). Nucleic Acids Res..

[32] Nishimura Y., Yoshida T., Kuronishi M., Uehara H., Ogata H., Goto S.J.B. (2017). ViPTree: The viral proteomic tree server. Bioinformatics.

[33] Abeysekera G.S., Love M.J., Manners S.H., Billington C., Dobson R.C.J. (2022). Bacteriophage-encoded lethal membrane disruptors: Advances in understanding and potential applications. Front. Microbiol..

[34] Hyman P.J.P. (2019). Phages for phage therapy: Isolation, characterization, and host range breadth. Pharmaceuticals.

[35] Ramesh N., Archana L., Madurantakam Royam M., Manohar P., Eniyan K.J.A.M. (2019). Effect of various bacteriological media on the plaque morphology of Staphylococcus and Vibrio phages. Access Microbiol..

[36] Kotz P., Fisher J., McCluskey P., Hartwell S.D., Dharma H. (2009). Use of a new silver barrier dressing, ALLEVYN? Ag in exuding chronic wounds. Int. Wound J..

[37] Pallavali R.R., Degati V.L., Lomada D., Reddy M.C., Durbaka V.R.P. (2017). Isolation and in vitro evaluation of bacteriophages against MDR-bacterial isolates from septic wound infections. PLoS ONE.

[38] Esebelahie N., Newton-Esebelahie F., Omoregie R. (2013). Aerobic bacterial isolates from infected wounds. Afr. J. Clin. Exp. Microbiol..

[39] Patil A., Banerji R., Kanojiya P., Koratkar S., Saroj S. (2021). Bacteriophages for ESKAPE: Role in pathogenicity and measures of control. Expert Rev. Anti-Infect. Ther..

[40] Herridge W.P., Shibu P., O’Shea J., Brook T.C., Hoyles L. (2020). Bacteriophages of Klebsiella spp., their diversity and potential therapeutic uses. J. Med. Microbiol..

[41] Chan B.K., Abedon S.T., Loc-Carrillo C. (2013). Phage cocktails and the future of phage therapy. Future Microbiol..

[42] Cano E.J., Caflisch K.M., Bollyky P.L., Van Belleghem J.D., Patel R., Fackler J., Brownstein M.J., Horne B.A., Biswas B., Henry M. (2021). Phage therapy for limb-threatening prosthetic knee Klebsiella pneumoniae infection: Case report and in vitro characterization of anti-biofilm activity. Clin. Infect. Dis..

[43] Yang L., Wang C., Zeng Y., Song Y., Zhang G., Wei D., Li Y., Feng J. (2024). Characterization of a novel phage against multidrug-resistant Klebsiella pneumoniae. Arch. Microbiol..

[44] Singh A., Singh A.N., Rathor N., Chaudhry R., Singh S.K., Nath G. (2022). Evaluation of bacteriophage cocktail on septicemia caused by colistin-resistant Klebsiella pneumoniae in mice model. Front. Pharmacol..

[45] Delbrück M. (1940). The growth of bacteriophage and lysis of the host. J. Gen. Physiol..

[46] Kolmogorov M., Bickhart D.M., Behsaz B., Gurevich A., Rayko M., Shin S.B., Kuhn K., Yuan J., Polevikov E., Smith T.P. (2020). metaFlye: Scalable long-read metagenome assembly using repeat graphs. Nat. Methods.

[47] Seemann T.J.B. (2014). Prokka: Rapid prokaryotic genome annotation. Bioinformatics.

[48] Arango-Argoty G., Garner E., Pruden A., Heath L.S., Vikesland P., Zhang L. (2018). DeepARG: A deep learning approach for predicting antibiotic resistance genes from metagenomic data. Microbiome.

[49] Naville M., Ghuillot-Gaudeffroy A., Marchais A., Gautheret D. (2011). ARNold: A web tool for the prediction of Rho-independent transcription terminators. RNA Biol..

