# Supplementary material for: Characterization and Genomic Analysis of a New Bacteriophage Klebsiella pneumoniae CTF-1 from Turkey
Source: Antibiotics (Basel). 2025 Nov 14;14(11):1153. doi: 10.3390/antibiotics14111153 (PMC12649526; doi:10.3390/antibiotics14111153)
Supplement: Supplementary file 1 [file antibiotics-14-01153-s001.zip › antibiotics-3946187-supplementary.pdf]

Table S1. Annotation of The *K. pneumoniae* CTF-1 phage genome

Prokka_name	name	acession	function	taxonomy	% identity	alignment length	mismatches	gap opens	q. start	q. end	evalue	bit score
PROKKA_00001	Gp1	UZT48329.1	DNA ligase	[Klebsiella phage vB_KpnP_K2044-302]	95.862	145	6	0	1	145	5.23E-98	293
PROKKA_00002	Gp2	YP_003347528.1	BC10 family protein	[Klebsiella phage KP32]	100	87	0	0	1	87	3.29E-57	178
PROKKA_00003	Gp3	YP_009966341.1	nucleotide kinase	[Klebsiella phage SH-Kp 152234]	85.075	134	11	1	1	125	1.38E-75	228
PROKKA_00004	Gp4	YP_009190966.1	hypothetical protein AU151_gp19	[Klebsiella phage vB_Kp1]	100	41	0	0	1	41	7.41E-23	88.2
PROKKA_00005	Gp5	YP_009801318.1	bacterial RNA polymerase inhibitor	[Klebsiella phage KP32_isolate 192]	100	49	0	0	1	49	2.33E-27	100
PROKKA_00006	Gp6	YP_009791668.1	single-stranded DNA-binding protein	[Klebsiella phage 2044-307w]	99.567	231	1	0	1	231	2.04E-167	468
PROKKA_00007	Gp7	YP_004678737.1	endonuclease I	[Escherichia phage K30]	100	149	0	0	1	149	1.38E-105	305
PROKKA_00008	Gp8	YP_009818012.1	N-acetylmuramoyl-L-alanine amidase	[Klebsiella phage KN3-1]	100	151	0	0	1	151	5.68E-111	320
PROKKA_00009	Gp9	YP_009791671.1	toprim domain-containing protein	[Klebsiella phage 2044-307w]	99.803	508	1	0	1	508	0	1046
PROKKA_00010	Gp10	YP_009818413.1	hypothetical protein HOU94_gp19	[Klebsiella phage Henu1]	98.551	69	1	0	1	69	5.14E-36	124
PROKKA_00011	Gp11	QAU05525.1	hypothetical protein D3A56_0020	[Klebsiella phage Kund-ULIP47]	100	90	0	0	1	90	5.48E-58	180
PROKKA_00012	Gp12	YP_009786867.1	hypothetical protein HOR24_gp23	[Klebsiella phage vB_KpnP_KpV766]	98.425	127	2	0	1	127	1.13E-87	258
PROKKA_00013	Gp13	YP_009821359.1	DNA polymerase	[Klebsiella phage Pharr]	98.729	708	9	0	1	708	0	1455

PROKKA_ 00014	Gp1 4	QFR5724 8.1	hypothetical protein AmPhEK52_0024	[Klebsiella phage AmPh_EK52]	99	100	1	0	1	10 0	5.98E -66	201
PROKKA_ 00015	Gp1 5	YP_0091 90979.1	HNS binding protein	[Klebsiella phage vB_Kp1]	100	69	0	0	1	69	1.21E -43	142
PROKKA_ 00016	Gp1 6	YP_0097 87480.1	hypothetical protein HOR32_gp34	[Klebsiella phage vB_KpnP_BIS33]	94.82 8	58	3	0	1	58	7.53E -34	117
PROKKA_ 00017	Gp1 7	YP_0098 17994.1	exonuclease	[Klebsiella phage KN4-1]	99.33 6	301	2	0	1	30 1	0	622
PROKKA_ 00018	Gp1 8	YP_0097 86824.1	hypothetical protein HOR23_gp32	[Klebsiella phage vB_KpnP_KpV767]	100	36	0	0	1	36	2.71E -15	68.9
PROKKA_ 00019	Gp1 9	YP_0020 03818.1	DUF2717 domain-containing protein	[Klebsiella phage K11]	98.76 5	81	1	0	1	81	4.15E -53	167
PROKKA_ 00020	Gp2 0	YP_0097 86826.1	DUF5476 domain-containing protein	[Klebsiella phage vB_KpnP_KpV767]	100	73	0	0	1	73	3.16E -44	144
PROKKA_ 00021	Gp2 1	QEQ5045 6.1	putative tail assembly protein	[Klebsiella phage vB_KpnP_IME335]	98.83 7	86	1	0	1	86	3.41E -51	162
PROKKA_ 00022	Gp2 2	QCG7644 8.1	portal protein	[Klebsiella phage vB_KpnP_FZ12]	100	535	0	0	1	53 5	0	108 7
PROKKA_ 00023	Gp2 3	YP_0098 01463.1	capsid assembly protein	[Klebsiella phage KP32_isolate 196]	98.12 5	320	6	0	1	32 0	0	638
PROKKA_ 00024	Gp2 4	YP_0098 17997.1	capsid and scaffold protein	[Klebsiella phage KN4-1]	100	343	0	0	1	34 3	0	696
PROKKA_ 00025	Gp2 5	YP_0099 66366.1	minor capsid protein	[Klebsiella phage SH-Kp 152234]	100	73	0	0	1	73	1.71E -44	145
PROKKA_ 00026	Gp2 6	YP_0091 98661.1	tail protein	[Klebsiella phage K5]	99.47 9	192	1	0	1	19 2	5.89E -141	398
PROKKA_ 00027	Gp2 7	YP_0098 01379.1	tail tubular protein B	[Klebsiella phage KP32_isolate 194]	99.11 5	791	7	0	1	79 1	0	162 7
PROKKA_ 00028	Gp2 8	QBG7838 1.1	internal (core) protein	[Klebsiella phage Kund-ULIP54]	99.26 5	136	1	0	1	13 6	6.99E -99	287
PROKKA_ 00029	Gp2 9	YP_0098 18001.1	internal virion protein B	[Klebsiella phage KN4-1]	99.49	196	1	0	1	19 6	1.24E -139	395

PROKKA_ 00030	Gp3 0	QOV0549 9.1	DNA ejectosome component	[Klebsiella phage P560]	99.46 7	751	4	0	1	75 1	0	153 6
PROKKA_ 00031	Gp3 1	QAU0554 4.1	peptidoglycan transglycosylase	[Klebsiella phage Kund-ULIP47]	98.78 9	1321	16	0	1	13 21	0	268 5
PROKKA_ 00032	Gp3 2	YP_0033 47555.1	tail fiber protein	[Klebsiella phage KP32]	95.37 3	670	31	0	1	67 0	0	130 3
PROKKA_ 00033	Gp3 3	YP_0033 47555.1	tail fiber protein	[Klebsiella phage KP32]	95.45 5	176	8	0	1	17 6	2.29E -113	349
PROKKA_ 00034	Gp3 4	DAH9086 9.1	MAG TPA: tailspike protein	[Myoviridae sp.]	48.07	570	287	7	34	59 5	2.02E -180	535
PROKKA_ 00035	Gp3 5	UVX2971 7.1	holin	[Klebsiella phage VLCpiA3a]	100	67	0	0	1	67	6.57E -40	133
PROKKA_ 00036	Gp3 6	YP_0097 86789.1	putative DNA packaging protein A	[Klebsiella phage vB_KpnP_KpV763]	100	85	0	0	1	85	4.31E -55	172
PROKKA_ 00037	Gp3 7	YP_0097 86842.1	lysis system i-spanin subunit Rz	[Klebsiella phage vB_KpnP_KpV767]	97.97 3	148	3	0	1	14 8	4.90E -102	296
PROKKA_ 00038	Gp3 8	QOV0639 9.1	hypothetical protein DNHFEEIH_00085	[Klebsiella phage 066013]	99.82 9	585	1	0	1	58 5	0	121 7
PROKKA_ 00039	Gp3 9	YP_0046 78769.1	gp19.5 family protein	[Escherichia phage K30]	100	49	0	0	1	49	1.59E -24	92.8
PROKKA_ 00040	Gp4 0	UMO770 70.1	S-adenosyl-L-methionine hydrolase	[Klebsiella phage IME264]	97.41 9	155	4	0	1	15 5	1.65E -109	315
PROKKA_ 00041	Gp4 1	QAU0550 7.1	hypothetical protein D3A56_0002	[Klebsiella phage Kund-ULIP47]	93.33 3	60	4	0	1	60	4.14E -36	122
PROKKA_ 00042	Gp4 2	QXO1045 7.1	hypothetical protein	[Klebsiella phage vB_KoxP_ZX8]	96.92 3	65	2	0	1	65	3.99E -38	128
PROKKA_ 00043	Gp4 3	QEG0995 1.1	hypothetical protein KMI2_21	[Klebsiella phage KMI2]	94.64 3	56	3	0	1	56	2.39E -28	103
PROKKA_ 00044	Gp4 4	UUU4538 0.1	kinase	[Klebsiella phage NK20]	91.57 6	368	31	0	1	36 8	0	643
PROKKA_ 00045	Gp4 5	UUU4537 9.1	DNA dependent RNA polymerase	[Klebsiella phage NK20]	99.55 8	906	4	0	1	90 6	0	187 8

PROKKA_ 00046	Gp4 6	YP_0098 17989.1	hypothetical protein HOU86_gp04	[Klebsiella phage KN4-1]	97.32 6	187	5	0	1	18 7	2.12E -131	374
PROKKA_ 00047	Gp4 7	YP_0099 66337.1	hypothetical protein HOT34_gp08	[Klebsiella phage SH-Kp 152234]	94.73 7	76	4	0	1	76	1.28E -45	148

Table S2. rho-independent terminator locations

Rho #	location	Motifs	strand	Sequence	Score
rho 1	2423	Rnamotif	+	GTGAGCAATACCCGGAGTTAGACATCCGGTTAGTGTTCTCT	-8.1
rho 2	4967	Both	+	GTAATCAGCAAGGGGACATGCCTAATCTTGTCCTCcTTCGTATTCTCA	-9.3
rho 3	14974	Both	+	TAATAAGCCAAACCCCTTGGGGACCACTCACGGTCTCTGAGGGGTTTTTTCGTTAGGA	-8.8
rho 4	25337	Rnamotif	+	GTAGCACTATAGGGAGACCGCTTGGTTTCCCTTCTAATTCAAC	-13.3
rho 5	30854	Rnamotif	+	CGCAACTGAAGGGTGACTTTGTCGCCTTTCTCTTCGTG	-7.4
rho 6	34663	Rnamotif	+	ACGCCCTGATTGTCCTGTACTTTGTGCGGGACTTTTGTAAGGGT	-10.5

Table S3: Tested antibiotics and mean zone diameters obtained for 25 *K. pneumoniae* strains

Antimicrobial agents	Mean zone diameter mm	EUCAST Zone diameter breakpoints for resistance mm	R %
Cefuroxime	6	<19	100
Ceftriaxon	6	<24	100
Ceftazidim	6	<19	100
Cefepim	6	<24	100
Amoxicillin-clavulanic acid	6	<19	100
Piperacillin-tazobactam	9	<20	100
Imipenem	10	<19	100
Meropenem	10	<16	100
Trimethoprim sulfamethoxazole	6	<11	100
Amikacin	11	<18	100
Tobramycin	10	<16	100
Ciprofloxacin	14	<22	100

Colistin	-	-	100

EUCAST: The European Committee on Antimicrobial Susceptibility Testing, R: Resistant
